# Anti-Tumor Necrosis Factor α Therapeutics Differentially Affect *Leishmania* Infection of Human Macrophages

**DOI:** 10.3389/fimmu.2018.01772

**Published:** 2018-07-31

**Authors:** Katharina Arens, Christodoulos Filippis, Helen Kleinfelder, Arthur Goetzee, Gabriele Reichmann, Peter Crauwels, Zoe Waibler, Katrin Bagola, Ger van Zandbergen

**Affiliations:** ^1^Division of Immunology, Paul-Ehrlich-Institut, Langen, Germany; ^2^Institute of Immunology, Johannes Gutenberg University, Mainz, Germany

**Keywords:** tumor necrosis factor α, remicade^®^, cimzia^®^, polyethylene glycol, leishmaniasis, complement, human macrophages, T-cells

## Abstract

Tumor necrosis factor α (TNFα) drives the pathophysiology of human autoimmune diseases and consequently, neutralizing antibodies (Abs) or Ab-derived molecules directed against TNFα are essential therapeutics. As treatment with several TNFα blockers has been reported to entail a higher risk of infectious diseases such as leishmaniasis, we established an *in vitro* model based on *Leishmania*-infected human macrophages, co-cultured with autologous T-cells, for the analysis and comparison of anti-TNFα therapeutics. We demonstrate that neutralization of soluble TNFα (sTNFα) by the anti-TNFα Abs Humira^®^, Remicade^®^, and its biosimilar Remsima^®^ negatively affects infection as treatment with these agents significantly reduces *Leishmania*-induced T-cell proliferation and increases the number of infected macrophages. By contrast, we show that blockade of sTNFα by Cimzia^®^ does not affect T-cell proliferation and infection rates. Moreover, compared to Remicade^®^, treatment with Cimzia^®^ does not impair the expression of cytolytic effector proteins in proliferating T-cells. Our data demonstrate that Cimzia^®^ supports parasite control through its conjugated polyethylene glycol (PEG) moiety as PEGylation of Remicade^®^ improves the clearance of intracellular *Leishmania*. This effect can be linked to complement activation, with levels of complement component C5a being increased upon treatment with Cimzia^®^ or a PEGylated form of Remicade^®^. Taken together, we provide an *in vitro* model of human leishmaniasis that allows direct comparison of different anti-TNFα agents. Our results enhance the understanding of the efficacy and adverse effects of TNFα blockers and they contribute to evaluate anti-TNFα therapy for patients living in countries with a high prevalence of leishmaniasis.

## Introduction

Tumor necrosis factor α (TNFα) is a pleiotropic, pro-inflammatory cytokine that mediates a diverse range of biologic effects. It is expressed as membrane-integrated form (mTNFα) or, upon cleavage by TNFα-converting enzyme, as soluble TNFα (sTNFα). TNFα signals through two receptors, membrane TNF receptor 1 (mTNFR1) and 2 (mTNFR2), which differ in structure, expression pattern, and activated downstream signaling pathways (1). Numerous studies implicate excessive levels of TNFα to contribute to the pathophysiology of human autoimmune diseases such as rheumatoid arthritis and inflammatory bowel disease of which millions of people are affected worldwide (1–3). Therapeutic antibodies (Abs), Ab fragments, and fusion proteins directed against TNFα have revolutionized treatment of TNFα-associated autoimmune diseases and are currently used with great success (1, 4). Remicade^®^, a chimeric murine-human IgG1 Ab, was the first anti-TNFα Ab approved by the European Medicines Agency (EMA) in 1999. Further TNFα blockers like the fully human Abs Humira^®^ and Simponi^®^, the TNFR2-fragment crystallizable (Fc) fusion protein Enbrel^®^ and the polyethylene glycol (PEG)-conjugated antigen-binding (Fab) fragment-derived inhibitor Cimzia^®^ followed in subsequent years (5). Patent expiration promoted the development of copy versions (biosimilars) by competitor companies. Accordingly, several biosimilars received marketing authorization in recent years or are currently under clinical investigation (4, 5). Although directed against the same target, TNFα blockers can differ in their mode of action as they are large and complex molecules with diverse structures (4, 6).

Besides its role in human autoimmune diseases, TNFα plays an important role in the control of infectious diseases such as tuberculosis or leishmaniasis (1, 7, 8). Leishmaniasis, which is endemic in tropical and subtropical regions, is caused by infection with the protozoan parasite *Leishmania*. Disease manifestations include cutaneous, mucosal, and visceral syndromes, depending on the parasite species and the host’s immune response (9, 10). More than one million new cases are estimated to occur annually with increased spreading of parasites to previously non-endemic countries (10, 11). Mouse models demonstrated disease promotion in *Leishmania*-infected mice upon neutralization of TNFα by Abs (12). Likewise, studies in humans revealed a correlation of *Leishmania* infection with TNFα. Increased expression of TNFα was found in cutaneous and mucosal lesions and TNFα levels were highly elevated in sera of patients during active disease. However, concentrations declined upon effective therapy of leishmaniasis (13–15). Immunosuppressive anti-TNFα therapy in humans is linked to a higher susceptibility for an infection with *Leishmania* or a reactivation of latent leishmaniasis (16–23), including reports that suggest differences in parasite control depending on the type of TNFα blocker applied (24–26). Similar to leishmaniasis, a higher incidence of tuberculosis has been described after anti-TNFα therapy. Clinical reports indicate that tuberculosis infections occur more frequently in patients treated with Remicade^®^ or Humira^®^ (27).

In the present study, we tested the hypothesis that therapeutic TNFα inhibitors, varying in their amino acid sequence or structure, differently influence *Leishmania major* (*Lm*) infection control. Focusing on human macrophages as *Lm* parasite reservoir and activated autologous T-cells as effector cells to combat parasites (9), we established an *in vitro* model representative for cutaneous leishmaniasis (10). We compared four different TNFα blockers by analyzing their effects on *Lm*-induced T-cell proliferation and *Lm* infection rates in macrophages. Our results show that blockade of sTNFα by Remicade^®^, Remsima^®^, and Humira^®^ strongly reduces activation of T-cells and consequently increases the number of *Lm*-infected macrophages. Neutralization of sTNFα by Cimzia^®^ does not interfere with T-cell effector function and *Lm* infection rates. We can link these diverging effects of Cimzia^®^ to PEG-induced activation of the complement system, which presumably contributes to maintain control of *Leishmania* parasites. Thus, we suggest that anti-TNFα therapy using Cimzia^®^ is potentially beneficial for patients living in high-risk areas of leishmaniasis.

## Materials and Methods

### Parasites

Wild-type or transgenic *Lm* promastigotes (MHOM/IL/81/FEBNI) expressing either a red (DsRed) or green fluorescent (EGFP) protein were obtained and cultured as described (28). For the infection of human macrophages, parasites of the stationary growth phase (6–8 days of cultivation) were used. These contain a higher proportion of apoptotic cells compared to parasites of the logarithmic growth phase (29).

### Cell Purification

Human peripheral blood mononuclear cells were isolated from buffy coats (DRK-Blutspendedienst Hessen GmbH, 506838) of healthy donors as described (30). If not indicated otherwise, monocytes were enriched by plastic adherence. Monocytes were cultivated (37°C, 5% CO_2_) in complete medium (CM) consisting of RPMI 1640 (Biowest) supplemented with 10% fetal calf serum (FCS, Sigma Aldrich), 50 µM β-mercaptoethanol (Sigma Aldrich), 2 mM l-glutamine, 100 U/mL penicillin, 100 µg/mL streptomycin, and 10 mM HEPES (*N*-2-hydroxyethylpiperazine-*N*′-2-ethanesulfonic acid) (all from Biochrom AG). For the generation of human monocyte-derived macrophages (hMDMs), 10 ng/mL recombinant human granulocyte-macrophage colony-stimulating factor (Bayer) were added for 5–7 days of cultivation. Separated monocytes were obtained by magnetic activated cell sorting (MACS) and CD14^+^ selection (CD14 MicroBeads, Miltenyi Biotec). Autologous peripheral blood lymphocytes (PBLs), which comprise 70–90% T-cells, were collected and stored frozen in CM containing 30% FCS and 10% dimethyl sulfoxide (DMSO, Sigma Aldrich). Untouched CD3^+^ or naive CD3^+^ T-cells were obtained using negative selection (Pan T-Cell Isolation Kit or Naive Pan T-Cell Isolation Kit, Miltenyi Biotec) after thawing of PBLs.

### Infection of Primary Human Macrophages and Co-Incubation with T-Cells

After 5–7 days of cultivation, adherent hMDMs were detached, counted (CASY) and 0.6 × 10^6^ hMDMs were seeded in 1.5 mL microcentrifuge tubes. For infection, 12 × 10^6^ *Lm* were added with a multiplicity of infection (MOI) = 20 and hMDMs were incubated at 37°C, 5% CO_2_. After 3 h, extracellular parasites were removed by washing hMDMs twice with CM. 24 h post-infection, hMDMs were distributed (0.1 × 10^6^ cells/tube) to enable longer cultivation. If necessary, Fcγ receptors (FcγRs) on hMDMs were saturated by pre-incubation (1 h, 37°C) with 20 µg/mL Polyglobin^®^ (Bayer) prior to distribution. Then, stored PBLs were thawed, counted (CASY), separated by MACS if necessary and labeled with CFSE [5-(and 6)-Carboxyfluorescein diacetate succinimidyl ester, Sigma] as described previously (30). Given that the hMDM culture still contains 1–4% lymphocytes, hMDMs and the remaining lymphocytes were also stained with CFSE prior to seeding. Excess CFSE was removed by washing cells with CM. For the PBL-based T-cell assay, 0.5 × 10^6^ PBLs and for the purified T-cell-based T-cell assay, 0.5 × 10^6^ separated T-cells were added to distributed hMDMs. Cells were incubated and analyzed either 24 h post-infection (hMDMs) or 7 days post-infection (hMDM/PBL co-culture).

### Neutralization of Cytokines

Therapeutic anti-TNFα agents were used in equimolar amounts and according to their ability to neutralize sTNFα as proven by an ELISA. Micrograms of TNFα inhibitors were calculated from the given molecular weights. Cells were treated with 20 µg/mL Remicade^®^ (infliximab, approximately 149 kDa, Janssen Biologics), 20 µg/mL Remsima^®^ (infliximab, approximately 149 kDa, Celltrion Healthcare), 20 µg/mL Humira^®^ (adalimumab, approximately 148 kDa, AbbVie), or 13 µg/mL Cimzia^®^ (certolizumab pegol, approximately 91 kDa including 2 × 20 kDa PEG, UCB). In contrast to the other TNFα blockers used here, Cimzia^®^ contains only one binding site for TNFα. We therefore determined the TNFα-neutralizing capacity of Cimzia^®^ by titration (Figure S1 in Supplementary Material). TNFα inhibitors were added to each microcentrifuge tube immediately after distribution of hMDMs and the addition of PBLs or T-cells.

### PEGylation

Primary amino (−NH_2_) groups of Remicade^®^ were PEGylated with 1.2 kDa MS-PEG (Methyl-PEG_24_-*N*-Hydroxysuccinimid-Ester, Thermo Scientific). Remicade^®^ was incubated with 20-fold molar excess of MS-PEG for 30 min at room temperature (RT) as recommended by the manufacturer. Equimolar amounts of PEG-Remicade^®^ and Remicade^®^ were then added to infected hMDMs as indicated above.

### Diff-Quik^®^ Staining

For microscopic analysis (Zeiss AxioPhot), at least 10^5^ hMDMs were sedimented on glass slides (Tharmac) by centrifugation at 75 *g* for 5 min. Afterward, slides were air-dried, fixed with methanol (2 min, RT), and stained (2 min, RT) using Diff-Quik^®^ solution I and II (Medion Diagnostics). Excess dye was washed away with water.

### Flow Cytometry

0.15–0.4 × 10^6^ cells were seeded in 96-well plates (Sarstedt). Samples were incubated for 5–10 min with 5 pg/mL propidium iodide (PI, Sigma Aldrich) before detecting dead cells (PI^+^). Proliferation of viable T-cells was determined by the reduction of CFSE (CFSE^low^) and *Lm* infection rates in viable hMDMs were assessed by DsRed- or EGFP-expressing *Lm* as previously described (30). For surface expression analysis, cells were stained with fluorescent-labeled Abs (Table S1 in Supplementary Material) or corresponding isotype controls as defined by the manufacturer. Intracellular analysis required fixation with 4% paraformaldehyde (Sigma Aldrich) for 10 min on ice and permeabilization with 0.5% saponin (Sigma Aldrich) prior to staining. Results were recorded using a BD LSR II SORP flow cytometer (BD Bioscience) and analyzed by FlowJo software (Tree Star). Gating strategies are depicted in Figure S2 in Supplementary Material. The mean fluorescence intensity (MFI) of specific markers was normalized to the respective isotype control by division and is presented as relative fluorescence intensity (RFI). For T-cell proliferation and *Lm* infection rates, values of the respective controls were subtracted for each donor and condition, respectively, which allowed donor-specific evaluation of different treatments.

### ELISA

24 h or 7 days after infection, cell culture supernatants were collected and frozen. They were thawed and analyzed for the presence of human sTNFα using R&D DuoSet ELISA according to the manufacturer’s protocol. Human C5a levels were examined with LEGEND MAX C5a ELISA Kit (BioLegend) according to the manufacturer’s instructions (exception: provide 20 µL Buffer B + 80 µL sample/standard and incubate at 4°C over night). Optical densities were measured with TECAN^®^ Infinite F50^®^ microplate reader and cytokine concentrations were determined by comparing optical densities to the respective standard curve (Microsoft^®^ Excel 2010).

### Statistical Analysis

All data are shown as mean ± SD. The number of independent experiments and donors (*n*) is depicted in each figure. Statistical significance was determined by the Wilcoxon signed-rank test (two-tailed, paired) using Graph-Pad Prism. “Not significant” is indicated as ns. A value of *P < 0.05; **P < 0.01; ***P < 0.001; and ****P < 0.0001 was considered statistically significant.

## Results

### Macrophages and T-Cells Differentially Express mTNFR1, mTNFR2, and TNFα

**Figure 1:**
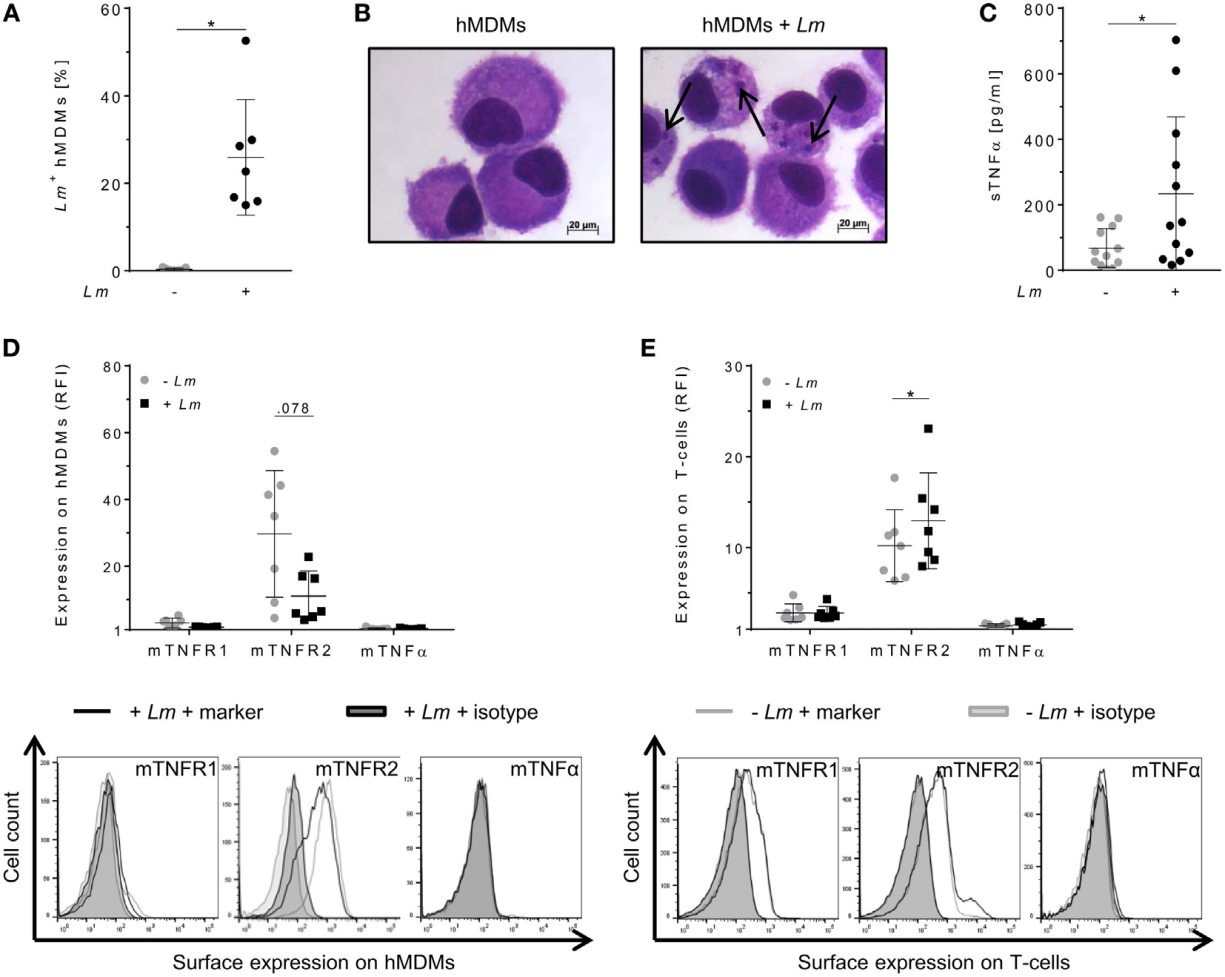
Expression of tumor necrosis factor α (TNFα) and mTNFRs by human monocyte-derived macrophages (hMDMs) or T-cells after *Leishmania major* (*Lm*) infection. Macrophages were infected with fluorescent *Lm* (multiplicity of infection = 20) and analyzed after 24 h. **(A)** Infection rates in hMDMs were determined by flow cytometry in comparison to non-infected hMDMs. **(B)** Infected and non-infected hMDMs were stained with Diff-Quik^®^ and representative micrographs are depicted. Black arrows indicate *Lm* parasites. **(C)** The concentration of soluble TNFα (sTNFα) in supernatants of hMDMs in the presence or absence of *Lm* was measured by ELISA 24 h post-infection. **(D)** Cell surface expression of mTNFR1, mTNFR2, or mTNFα on hMDMs was analyzed by flow cytometry 24 h after infection. **(E)** Peripheral blood lymphocytes were co-incubated with infected/non-infected hMDMs and cells were measured by flow cytometry after 7 days, with T-cells being defined by anti-CD3 Ab co-staining. **(D,E)** Relative fluorescence intensity was measured as the ratio of the mean fluorescence intensity (MFI) of specific markers to the MFI of isotype controls. Histograms show expression on hMDMs or T-cells in the non-infected (gray line) or infected culture (black line) in comparison to the isotype controls (black/gray solid). Data are presented as mean ± SD (*n* ≥ 7) and were obtained from at least two independent experiments. The Wilcoxon signed-rank test was performed to evaluate statistical significance. *P < 0.05.

To evaluate the relevance of TNFα-mediated signaling in our *in vitro* model, we assessed TNFα and mTNFR expression by primary hMDMs (host cells) and co-cultured autologous T-cells (effector cells) after infection of macrophages with *Lm*. Infection was confirmed by flow cytometry (Figure 1A) as well as by Diff-Quik^®^ stained cytospins (Figure 1B). Thereafter, we measured the amount of secreted sTNFα in supernatants of hMDMs by ELISA. In line with previous reports (30–33), release of sTNFα by hMDMs after *Lm* infection was significantly increased (234 ± 235 vs 68 ± 59 pg/mL) (Figure 1C). Cell surface expression of mTNFR1, mTNFR2, and mTNFα on hMDMs was determined as RFI using flow cytometry. Gating strategies for flow cytometric analyses are depicted in Figure S2 in Supplementary Material. In order to investigate the influence of *Lm* infection on mTNFR and mTNFα surface expression on T-cells, we co-incubated infected or non-infected hMDMs with autologous PBLs comprising 70–90% T-cells (Figure S3 in Supplementary Material). We detected that hMDMs and T-cells more frequently expressed mTNFR2 than mTNFR1 on their cell surface (Figures 1D,E). *Lm* infection slightly reduced mTNFR2 expression on hMDMs (mean RFI: 11 ± 7 vs 30 ± 19), whereas mTNFR2 levels were significantly increased on T-cells (mean RFI: 13 ± 5 vs 10 ± 4) after co-incubation with *Lm*-infected hMDMs. Surface expression of mTNFα was not detected on hMDMs and T-cells, neither in the absence nor in the presence of an infection with *Lm* (Figures 1D,E).

### CD4^+^ T-Cell Proliferation Reduces the Number of *Lm*-Infected Macrophages

**Figure 2:**
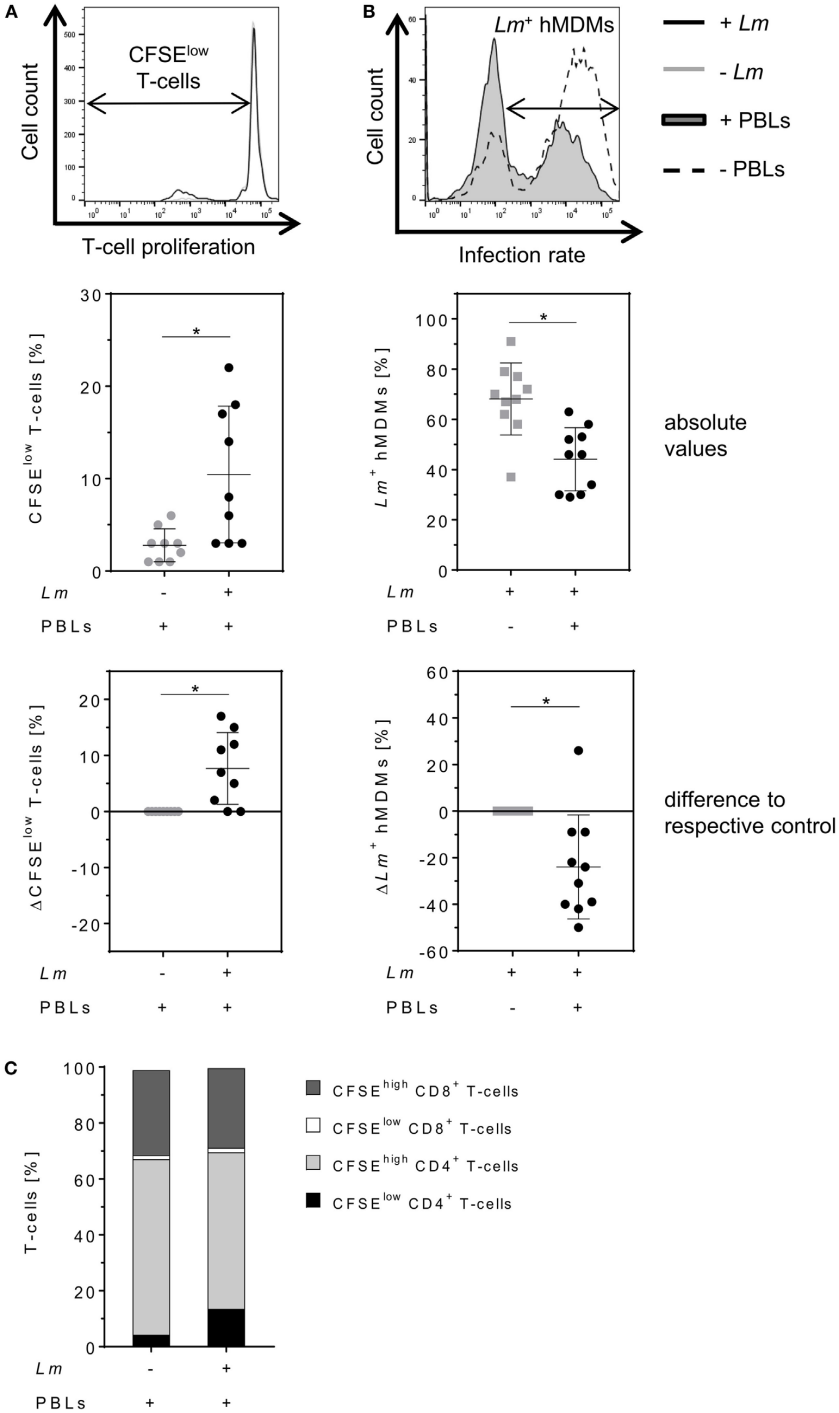
Proliferation of CD4^+^ T-cells reduces the number of *Leishmania major* (*Lm*)-infected macrophages. **(A,B)** *Lm*-infected/non-infected human monocyte-derived macrophages (hMDMs) were incubated in the absence or presence of CFSE-labeled autologous peripheral blood lymphocytes (PBLs). 7 days post-infection, T-cell proliferation (−*Lm* 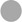, +*Lm* ●) and infection rates (−PBLs 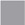, +PBLs ●) were measured by flow cytometry with T-cells being defined by anti-CD3 Ab co-staining. T-cell proliferation **(A)** and infection rates **(B)** are illustrated as representative histograms (upper panels), raw data (middle panels), or as differences by subtracting the respective controls from each corresponding donor (lower panels). **(C)** CD4/CD8 subset distribution was determined for proliferating (CFSE^low^) and non-proliferating (CFSE^high^) CD3^+^ T-cells in *Lm*-infected/non-infected hMDM/PBL co-cultures after 7 days. At least three independent experiments were conducted of which data are presented as mean ± SD (*n* ≥ 8). To determine statistical significance, the Wilcoxon signed-rank test was performed. *P < 0.05.

Parasite control in human cutaneous leishmaniasis is associated with *Leishmania*-induced T-cell activation which, in turn, stimulates macrophages to kill intracellular *Leishmania* (30, 34–36). We studied the impact of T-cells on *Lm*-infected hMDMs by flow cytometry analysis 6 days after hMDM/PBL co-culture. Previous studies in our lab revealed this time frame to be required for multiple T-cell divisions and for determining changes in T-cell proliferation (30). A significant induction of T-cell proliferation (10 ± 7%) in response to *Lm* infection of hMDMs compared to non-infected controls (3 ± 2%) could be observed (Figure 2A, middle panel). As a consequence, the percentage of *Lm*-infected hMDMs in the presence of PBLs (44 ± 13%) was significantly reduced compared to their absence (68 ± 14%) (Figure 2B, middle panel). This revealed a mean increase of T-cell proliferation of +8 ± 6% and a reduction of the infection rate of −24 ± 22% in PBL co-cultures when comparing corresponding donor values (Figures 2A,B, lower panel). Additional phenotypic characterization of CD3^+^ T-cells revealed that *Lm*-induced proliferating (CFSE^low^) T-cells expressed CD4 (14 ± 13% of total T-cell population). Only 2 ± 1% of total T-cells expressed CD8 and proliferated upon *Lm* infection. The percentage of non-proliferating (CFSE^high^) CD4^+^ T-cells in the *Lm*-infected co-culture was 56 ± 10% and the proportion of CFSE^high^ CD8^+^ T-cells was 28 ± 9% (Figure 2C).

### Anti-TNFα Therapeutics Similarly Neutralize sTNFα but Differentially Affect *Leishmania* Infection Control

**Figure 3:**
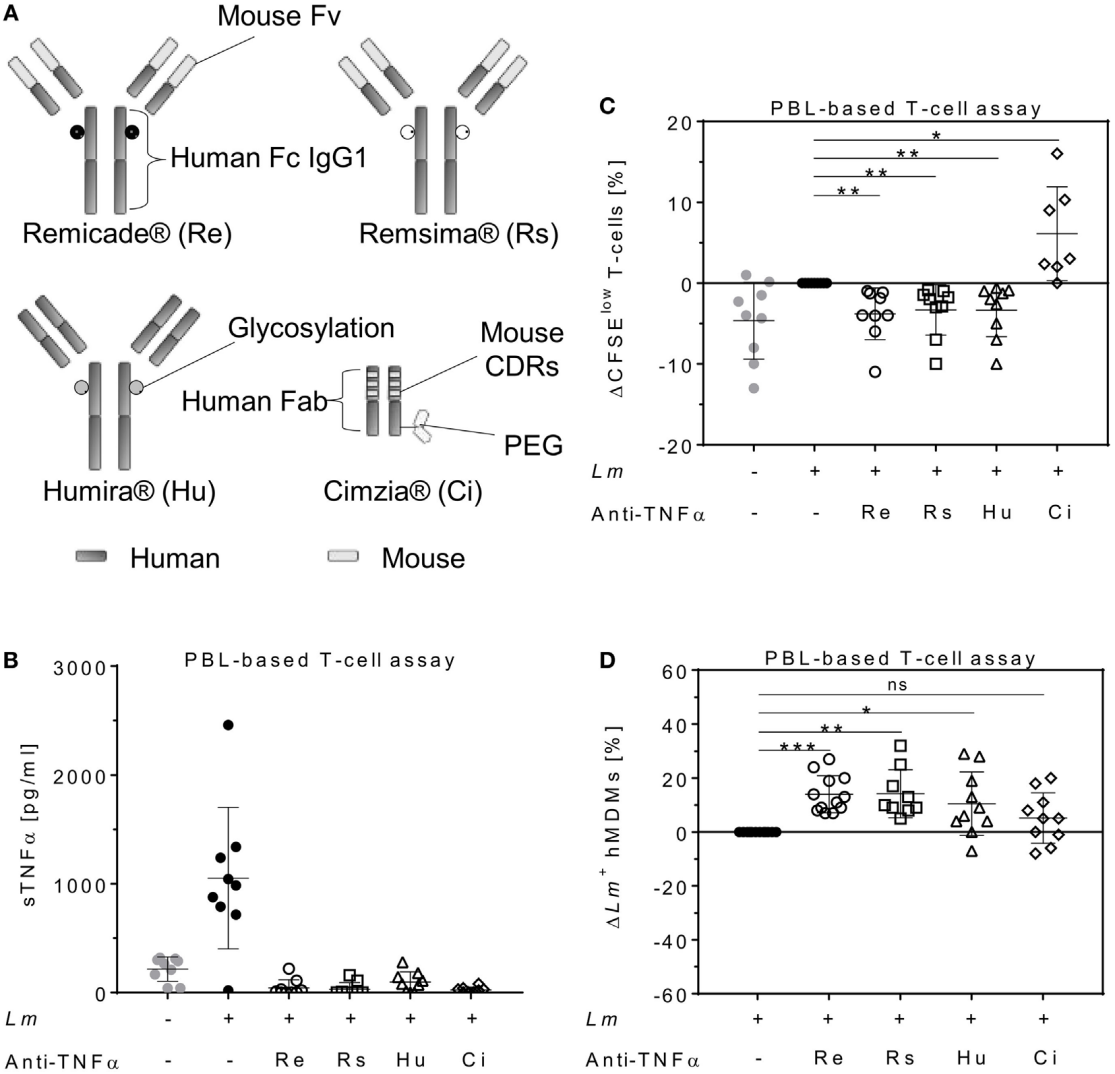
TNFα blockers have diverging effects on T-cell proliferation and *Lm* infection rates. **(A)** Schematic representation of the TNFα blockers Remicade^®^ (Re), Remsima^®^ (Rs), Humira^®^ (Hu), and Cimzia^®^ (Ci). **(B–D)** *Lm* (MOI = 20) were added to hMDMs for 3 h. Subsequently, extracellular parasites were removed by washing. Anti-TNFα agents (Re ○, Rs □, Hu Δ and Ci ◊) were added 24 h after *Lm* infection (−*Lm* 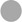, +*Lm* ●) of hMDMs and incubated in the presence of CFSE-labeled autologous PBLs. **(B)** The concentration of sTNFα was measured by ELISA 7 days post-infection. T-cell proliferation **(C)** and *Lm* infection rates in hMDMs **(D)** were analyzed by flow cytometry after 7 days. **(C,D)** Values of the corresponding controls were subtracted for each donor and condition to illustrate differences. Results are presented as mean ± SD (*n* ≥ 7) and at least four separate experiments were conducted. The Wilcoxon signed-rank test was performed to evaluate statistical significance. *P < 0.05; **P < 0.01; ***P < 0.001. Abbreviations: CDRs, complementarity determining regions; Fv, fragment variable; TNFα, tumor necrosis factor α; Lm, Leishmania major; MOI, multiplicity of infection; hMDMs, human monocyte- derived macrophages; PBLs, peripheral blood lymphocytes; sTNFα, soluble TNFα.

**Figure 4:**
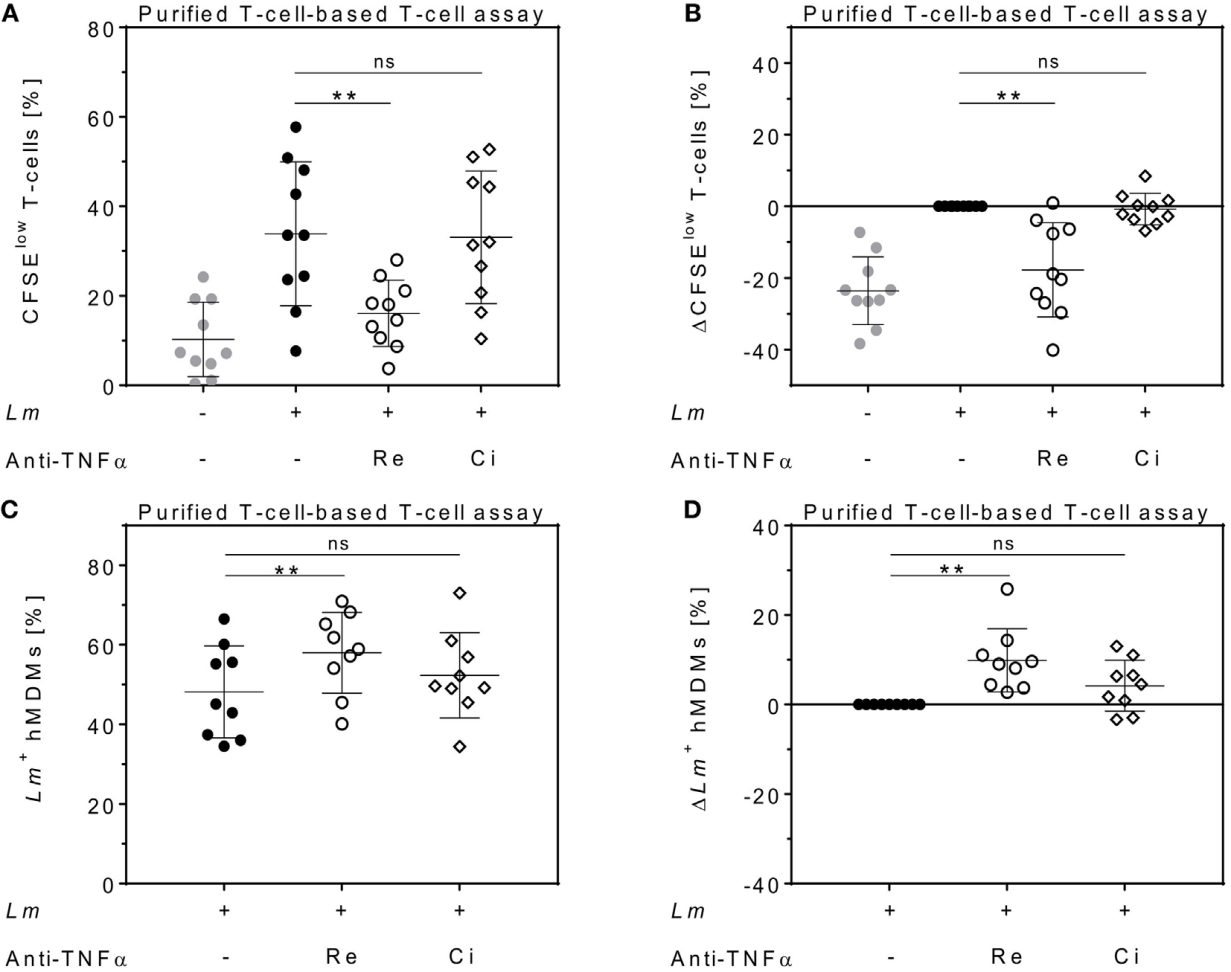
Effects of anti-tumor necrosis factor α (TNFα) agents are human monocyte-derived macrophage (hMDM)- and T-cell-mediated. Magnetic activated cell sorting-separated CD14^+^ hMDMs were infected with *Leishmania major* (*Lm*) (−*Lm* 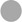, +*Lm* ●) and co-cultured with untouched CD3^+^ T-cells. The anti-TNFα agents Remicade^®^ (Re ○) and Cimzia^®^ (Ci ◊) were added and after 7 days, T-cell proliferation **(A,B)** and *Lm* infection rates in hMDMs **(C,D)** were analyzed by flow cytometry. **(A,C)** Raw data of T-cell proliferation and *Lm* infection rates are depicted without subtracting values from controls. **(B,D)** Values of the respective controls were subtracted for each donor and condition to illustrate differences. Results are presented as mean ± SD (*n* ≥ 9) and at least four separate experiments were conducted. The Wilcoxon signed-rank test was performed to evaluate statistical significance. ns *P* > 0.05; **P < 0.01.

To determine the impact of different TNFα blockers on *Leishmania* infection, we compared the chimeric Ab Remicade^®^, its biosimilar Remsima^®^, the fully human Ab Humira^®^, and the PEGylated Fab-derived inhibitor Cimzia^®^ (Figure 3A). First, anti-TNFα agents were tested by ELISA for their ability to neutralize sTNFα in the hMDM/PBL co-culture. *Lm* infection-induced sTNFα (1,053 ± 650 vs 216 ± 111 pg/mL) was effectively neutralized by all TNFα inhibitors and little or no sTNFα was measurable in cell culture supernatants (Figure 3B). We then determined proliferation of T-cells and the percentage of infected hMDMs by flow cytometry. *Lm*-induced T-cell proliferation was significantly reduced in the presence of Remicade^®^ (−4 ± 3%), Remsima^®^ (−3 ± 3%), and Humira^®^ (−3 ± 3%) (Figure 3C). Concomitantly, the percentage of *Lm*-infected hMDMs increased significantly (Remicade^®^: +14 ± 7%, Remsima^®^: +14 ± 9%, Humira^®^: +11 ± 12%) (Figure 3D). Remarkably, blockade of sTNFα by Cimzia^®^ did not reduce but significantly increase T-cell proliferation (+6 ± 6%) compared to non-treated controls (Figure 3C). Moreover, *Lm* infection rates in hMDMs did not change significantly (+5 ± 9%) after treatment with Cimzia^®^ (Figure 3D). Of note, we also measured *Lm* infection rates in the absence of PBLs, which, however, showed no significant differences after treatment with either of the four TNFα blockers compared to non-treated controls (Figure S4 in Supplementary Material). To assess whether diverging effects of Cimzia^®^ in the hMDM/PBL co-culture were exclusively hMDM- and T-cell-dependent, we examined the effects of TNFα blockers on *Lm* infection using MACS-purified CD14^+^ hMDMs and CD3^+^ T-cells. Focusing on Cimzia^®^ and Remicade^®^, we confirmed the previously obtained findings that, in contrast to Remicade^®^, Cimzia^®^ does not dampen T-cell proliferation (Figures 4A,B) and does not adversely influence *Lm* infection rates (Figures 4C,D). Inconsistent with the PBL-based T-cell assay, no increase in T-cell proliferation was observed after treatment with Cimzia^®^.

### Remicade^®^ but Not Cimzia^®^ Leads to Downregulation of Cytolytic Proteins in Proliferating T-Cells

**Figure 5:**
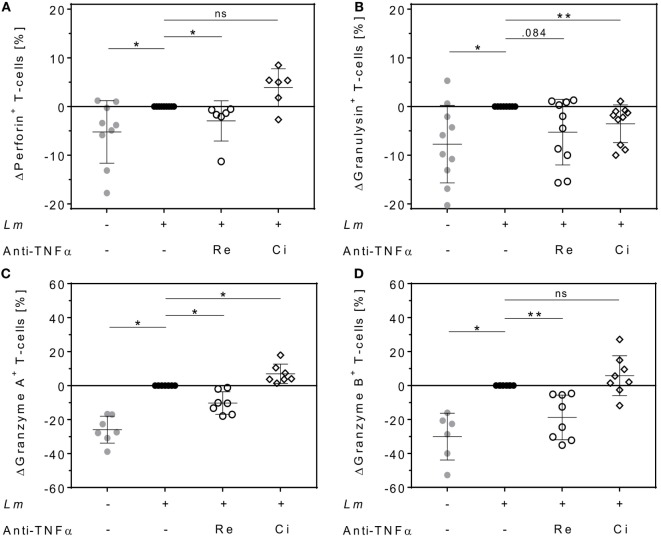
Intracellular expression of cytolytic proteins in proliferating CD4^+^ T-cells differs after soluble TNFα (sTNFα) neutralization by Remicade^®^ or Cimzia^®^. Non-infected (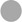) or *Leishmania major* (*Lm*)-infected human monocyte-derived macrophages (hMDMs) (●) were co-incubated with CFSE-labeled autologous peripheral blood lymphocytes and sTNFα was neutralized by Remicade^®^ (Re ○) or Cimzia^®^ (Ci ◊). Proliferating CD4^+^ T-cells expressing perforin **(A)**, granulysin **(B)**, granzyme A **(C)**, or granzyme B **(D)** were detected using anti-CD3 and anti-CD4 Ab co-staining in intracellular flow cytometry 7 days post-infection. Values are illustrated as differences to the untreated control of the same donor. Data are presented as mean ± SD (*n* ≥ 6) and were obtained in at least three independent experiments. To analyze statistical significance, the Wilcoxon signed-rank test was performed. *P < 0.05; **P < 0.01.

Cytolytic proteins such as perforin, granulysin, and granzymes are required for protective immunity against intracellular pathogens. They are expressed in effector T-cells and released upon antigen stimulation (37). We quantified intracellular levels of perforin, granulysin, granzyme A, and granzyme B in proliferating T-cells and assessed the effect of either Remicade^®^ or Cimzia^®^ treatment by flow cytometry. Expression of perforin (+5 ± 6%), granulysin (+8 ± 8%), granzyme A (+26 ± 8%), and granzyme B (+30 ± 14%) was significantly upregulated in proliferating CD4^+^ T-cells in the *Lm*-infected co-culture compared to non-infected controls (Figures 5A–D). This upregulation was largely reversed after neutralization of sTNFα by Remicade^®^, with perforin and granzymes being significantly reduced compared to non-treated controls (perforin: −3 ± 4%, granulysin: −5 ± 7%, granzyme A: −10 ± 7%, and granzyme B: −19 ± 13%) (Figures 5A–D). By contrast, neutralization of sTNFα by Cimzia^®^ did not interfere with the *Lm*-induced upregulation of perforin (+4 ± 4%), granzyme A (+7 ± 6%), and granzyme B (+6 ± 12%) (Figures 5A,C,D). Only granulysin levels (−4 ± 4%) were lowered and comparable to those measured after treatment with Remicade^®^ (Figure 5B). Raw data obtained for the expression of cytolytic molecules are depicted in Figure S5 in Supplementary Material. In this assay, the overall number of proliferating CD8^+^ T-cells that expressed cytolytic proteins was very low (<10%) and neutralization of sTNFα had no impact on cytolytic protein expression in these cells (data not shown). To assess whether treatment with Remicade^®^ or Cimzia^®^ affected viability of hMDMs and T-cells, the percentage of dead cells in the co-culture was determined with PI by flow cytometry. Altogether, the mean of PI^+^ T-cells and PI^+^ hMDMs was low and it ranged from 11 to 18% (Figure S6 in Supplementary Material). Values were comparable in non-treated and Remicade^®^- or Cimzia^®^-treated co-cultures, which demonstrates that sTNFα blockade does not affect host and effector cell viability.

### Differences Between Cimzia^®^ and Remicade^®^ Are Independent of Fc–FcγRs Interactions

**Figure 6:**
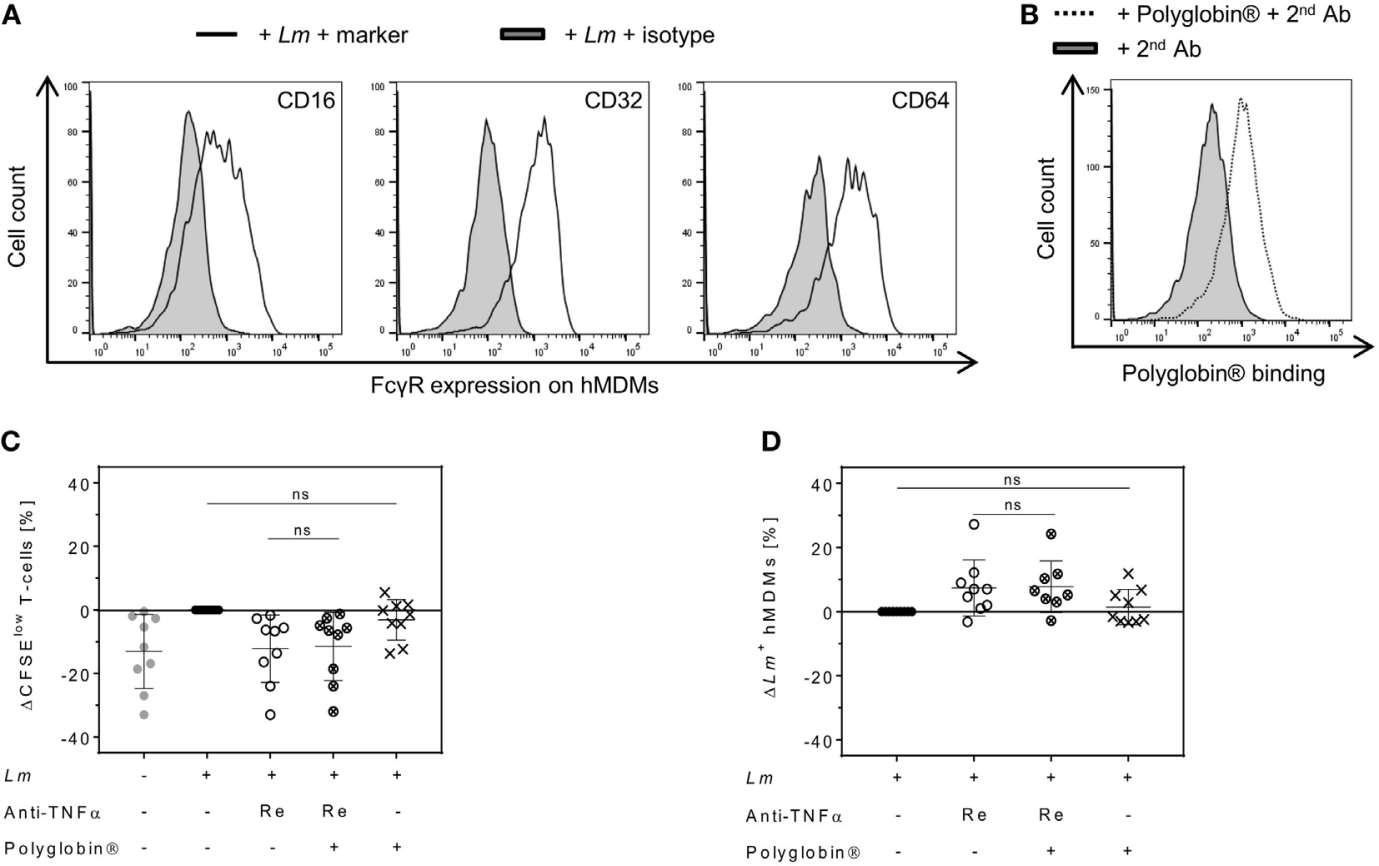
Reduced T-cell proliferation and increased *Leishmania major* (*Lm*) infection rates upon soluble TNFα (sTNFα) blockade by Remicade^®^ are independent of fragment crystallizable (Fc)–Fcγ receptor (FcγR) interactions. **(A)** Representative histograms of flow cytometry analysis show FcγR expression (black line) on infected human monocyte-derived macrophages (hMDMs) in comparison to the isotype control (black solid) 24 h after *Lm* infection. **(B)** 7 days after infection, binding of Polyglobin^®^ Abs to FcγRs on *Lm*-infected and Polyglobin^®^-treated hMDMs (black dotted line) in comparison to non-treated cells (black solid) was confirmed by flow cytometry using anti-human IgG staining. **(C,D)** *Lm*-infected (●) or non-infected (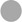) macrophages were pre-incubated with or without Polyglobin^®^ after which peripheral blood lymphocytes and Remicade^®^ (Re) were added (Remicade^®^ ○; Polyglobin^®^ ×; Remicade^®^ + Polyglobin^®^ ⊗). **(C)** T-cell proliferation and **(D)** infection rates in hMDMs of co-cultures were assessed by flow cytometry 7 days post-infection. Values are illustrated as differences to the untreated control of the same donor. Results are presented as mean ± SD (*n* ≥ 8) and were obtained from at least three independent experiments. Statistical analysis was carried out using the Wilcoxon signed-rank test. ns *P* > 0.05.

In contrast to the Fab-derived drug Cimzia^®^, Remicade^®^ is capable of binding to FcγRs *via* its Fc region (6). Signaling through FcγRs can alter cell activation and consequently might influence T-cell proliferation or *Lm* infection rates (6, 38). High expression of the FcγRs CD16, CD32, and CD64 on *Lm*-infected hMDMs was detected by flow cytometry (Figure 6A). To exclude that Fc–FcγR interactions of Remicade^®^ influenced T-cell proliferation or *Lm* infection rates, we saturated FcγRs on hMDMs by pre-incubation with the IgG preparation Polyglobin^®^ (39). Blockade of FcγRs was confirmed by flow cytometry (Figure 6B). We found that neutralization of sTNFα by Remicade^®^ equally reduced T-cell proliferation in the presence (−11 ± 11%) or absence of Polyglobin^®^ (−12 ± 11%) (Figure 6C). Likewise, *Lm* infection rates increased in the presence of Remicade^®^ with (+8 ± 8%) or without (+7 ± 9%) FcγR blockade (Figure 6D). Pre-incubation with Polyglobin^®^ devoid of sTNFα blockade showed no significant impact on T-cell expansion (−3 ± 6%) and the number of infected hMDMs (+1 ± 5%) in comparison with non-treated controls (Figures 6C,D). Thus, effects mediated by Remicade^®^ do not depend on Fc–FcγR interactions and suggest a different feature to be responsible for the diverging effects of Cimzia^®^.

### PEGylation of Remicade^®^ Increases T-Cell Proliferation and Reduces *Lm* Infection Rates

**Figure 7:**
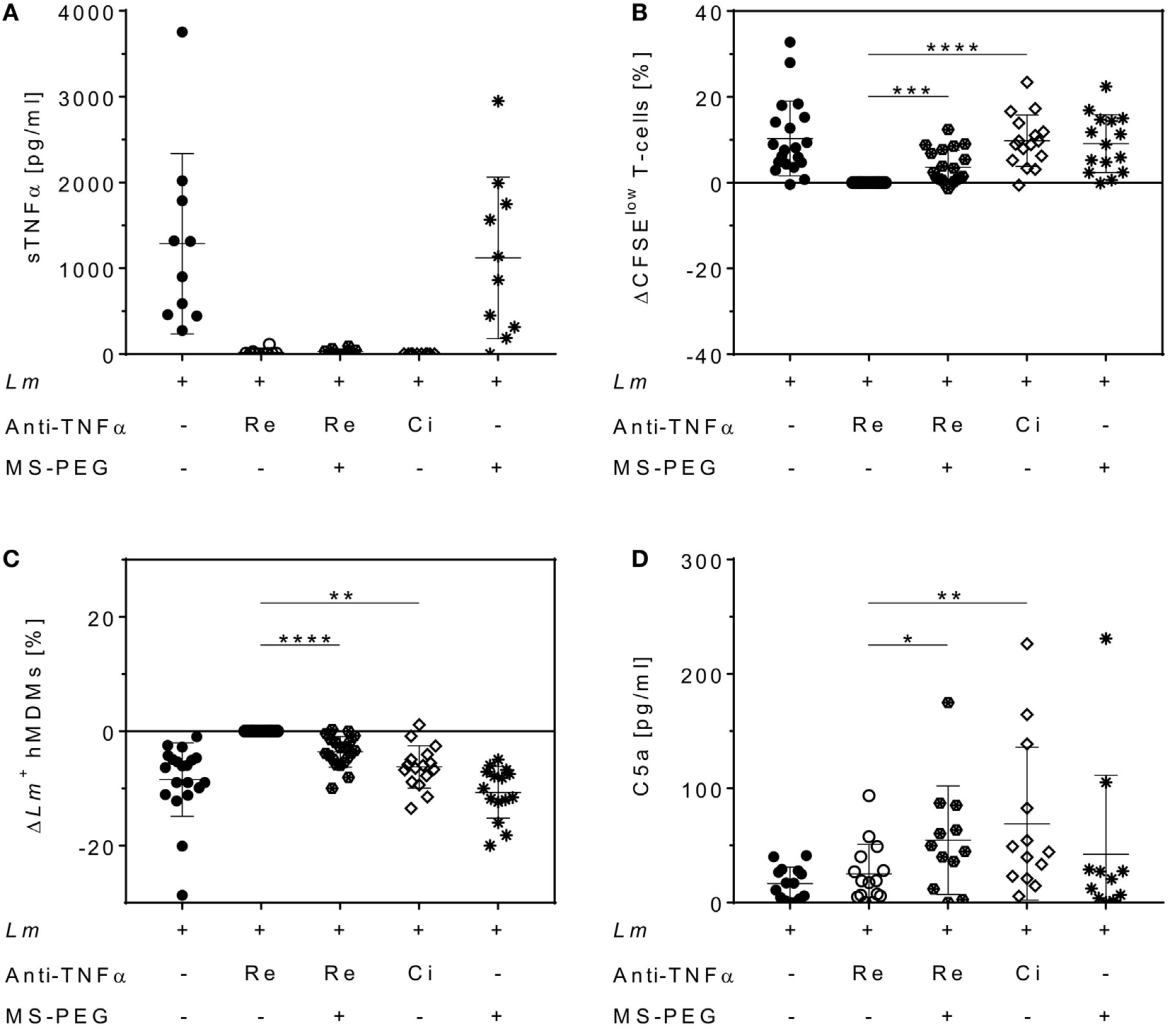
Increased T-cell proliferation and reduced *Leishmania major* (*Lm*) infection rates in human monocyte-derived macrophages (hMDMs) after PEGylation of Remicade^®^. The *Lm*-infected hMDM/peripheral blood lymphocyte co-culture (●) was treated with Remicade^®^ (○), PEGylated Remicade^®^ (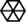), Cimzia^®^ (◊), and MS-polyethylene glycol (PEG) (∗). 7 days after infection, soluble TNFα (sTNFα) neutralization **(A)** or C5a levels **(D)** were measured by ELISA, and T-cell proliferation **(B)** as well as the percentage of *Lm*-infected hMDMs **(C)** were determined by flow cytometry. **(B,C)** T-cell proliferation and infection rates are presented as differences to the untreated control of the same donor. At least four independent experiments were conducted of which data are shown as mean ± SD (*n* ≥ 10). The Wilcoxon signed-rank test was performed to evaluate statistical significance. *P < 0.05; **P < 0.01; ***P < 0.001; ****P < 0.0001.

Polyethylene glycol can be added to therapeutic proteins to increase stability (40). We finally addressed the question whether a PEG moiety, present in Cimzia^®^ but not in Remicade^®^, could explain the differences in T-cell proliferation and *Lm* infection rates in hMDMs between these drugs. To this end, Remicade^®^ was PEGylated with the amine-reactive MS-PEG and the resulting PEG-Remicade^®^ or Cimzia^®^ was compared to the non-PEGylated form of Remicade^®^. Co-culture supernatants proved effective neutralization of sTNFα by all tested anti-TNFα agents by ELISA (Figure 7A). Subsequently, T-cell proliferation and *Lm* infection rates in hMDMs were evaluated by flow cytometry. Similar to Cimzia^®^ (+10 ± 6%), PEG-Remicade^®^ (+4 ± 4%) showed a significantly higher T-cell proliferation (Figure 7B) compared to non-PEGylated Remicade^®^. Likewise, the percentage of *Lm*-infected hMDMs was significantly reduced after treatment with Cimzia^®^ (−6 ± 4%) or PEG-Remicade^®^ (−4 ± 3%) (Figure 7C), demonstrating that PEG has a direct effect on T-cell proliferation and consequently *Lm* infection rates in hMDMs. As reported previously, PEG is able to activate complement (41). Therefore, we characterized the capacity of PEGylated TNFα inhibitors to activate the complement system by measuring the release of C5a, a marker of terminal complement activation (42). Remarkably, C5a levels were significantly higher in the presence of Cimzia^®^ (69 ± 67 pg/mL) compared to non-PEGylated Remicade^®^ (25 ± 26 pg/mL). Accordingly, the comparison of PEGylated with non-PEGylated Remicade^®^ demonstrated a significantly increased release of C5a (55 ± 47 pg/mL) after treatment with PEG-Remicade^®^ (Figure 7D). Altogether, these data link complement-mediated immunostimulation with the PEG moiety of Cimzia^®^ or PEG-Remicade^®^.

## Discussion

Although TNFα blockers have revolutionized therapy of autoimmune diseases, one of their major adverse effects is the significant risk of serious infections (6–8). Several reports link a higher incidence of leishmaniasis to the treatment with Remicade^®^, Humira^®^, Enbrel^®^, and Simponi^®^ (16–23). Therefore, we developed an *in vitro* model to investigate the impact of currently marketed therapeutic TNFα inhibitors on *Leishmania* infection.

After infection of macrophages with *Lm* and co-culture with autologous PBLs, we found an increased release of sTNFα into cell supernatants. Concomitantly, we observed an induction of CD4^+^ T-cell proliferation that, in agreement to previous reports, enhanced parasite control (30, 35). By determining the percentage of infected macrophages, a decreased number of hMDMs containing *Lm* was found upon PBL addition. The parasite burden per cell was not determined. Measured sTNFα levels and T-cell proliferation strongly differed among the tested donors, which might arise from the genetic diversity of human individuals.

We demonstrate the relevance of sTNFα for parasite control in human leishmaniasis and the negative impact of various anti-TNFα agents as treatment with Remicade^®^, Remsima^®^, and Humira^®^ increased infection rates in human macrophages. This increase resulted from reduced T-cell activation and proliferation. Although the differences in infection rate and T-cell proliferation seem to be not very high, the results we obtained are significant and effectual for almost every tested donor.

In agreement with our data, infection is a major adverse effect of immunosuppressive anti-TNFα treatment and reactivation of leishmaniasis or a higher susceptibility for an initial infection with *Leishmania* parasites has been linked to the application of several TNFα blockers by clinical reports (16–23). It is worth mentioning that the anti-TNFα agents tested in our experiments had no effect on *Lm* infection rates in the absence of PBLs, which illustrates the importance of T-cell activation for parasite control in humans. This result from our human model is in line with data obtained from *Lm* amastigote-infected peritoneal mouse macrophages (43). Here, treatment of the infected macrophages with various concentrations of mouse recombinant TNFα failed to activate macrophages for the killing of intracellular *Leishmania* and to reduce the initial infection rate.

Unlike the other anti-TNFα agents examined in our study, treatment with Cimzia^®^ maintained T-cell proliferation and parasite control despite effective sTNFα blockade. Furthermore, the overall expression of cytolytic molecules in proliferating CD4^+^ T-cells was not reduced by Cimzia^®^, demonstrating that treatment with this TNFα inhibitor does not interfere with T-cell effector functions. Surprisingly, levels of granulysin were lowered after Cimzia^®^ treatment compared to non-treated controls. Although granulysin has been described to have a direct antimicrobial effect (44, 45), parasite control was not affected in Cimzia^®^-treated samples. Here, other cytolytic proteins might compensate for the lower expression of granulysin, or a different effector mechanism might be initiated by Cimzia^®^.

Cytolytic proteins play a pivotal role in combating intracellular infections. They are released by activated T-cells, leading to pore formation in target cell membranes and promoting target cell lysis by not yet fully understood mechanisms (37). Of note, our experiments reveal increased levels of cytolytic molecules in *Lm*-induced proliferating CD4^+^ T-cells, although antimicrobial and cytotoxic activity has mainly been attributed to CD8^+^ T-cells (46, 47). In line with recent studies showing that CD4^+^ T-cells can also display cytolytic functions (47–49), we assume that cytolytic proteins released by CD4^+^ T-cells contribute to clearance of *Leishmania*. Dotiwala et al. demonstrated killing of intracellular parasites by cytolytic molecules independently of host cell death (45).

The role of cytolytic proteins in human leishmaniasis is controversial. Expression of granzyme A and granzyme B positively correlated with lesion progression in patients (34). In our study, *Lm*-infected hMDMs and co-cultured T-cells displayed low and comparable PI positivity in the absence or presence of anti-TNFα agents, demonstrating that cytolytic molecules expressed by CD4^+^ T-cells do not impair cell viability.

Among the tested anti-TNFα agents, Cimzia^®^ was the only one being modified with a PEG moiety. PEG is commonly used to increase half-life and stability and to reduce immunogenicity as well as aggregation of therapeutic proteins (40). It is described to have no adverse biological effects, although several studies revealed unanticipated immunogenicity of PEG (50).

We PEGylated Remicade^®^ to determine effects that might be caused by the PEG moiety. Our investigations indeed confirm an immunostimulatory effect of PEG as treatment with PEG-Remicade^®^ significantly increased T-cell proliferation and parasite control in infected macrophages. Several reports describe the development of anti-PEG Abs and activation of complement by PEG, though the underlying mechanisms and involved factors remain elusive (41, 42, 51–55). Furthermore, *in vitro* studies with human T-cells, co-cultured with allogeneic DCs, revealed that recombinant C3a and C5a promote CD4^+^ T-cell expansion (56). In fact, we found elevated levels of C5a in cell supernatants upon treatment with PEGylated Remicade^®^ or Cimzia^®^, which links complement activation with *Lm* infection control in the absence of sTNFα. Complement activation depends on the concentration and molecular weight of PEG (55). Thus, variations in the structure or size of the PEG moiety might be cause of the deviations between PEG-Remicade^®^ and Cimzia^®^.

Undesirable activation of the immune or complement system by PEG can result in clearance of PEGylated pharmaceuticals or potential hypersensitivity reactions (41). However, Cimzia^®^ demonstrated efficacy in clinical trials and is effectively and generally well tolerated used in therapy for almost 10 years (57).

The complement system has been traditionally regarded as innate system that controls invading pathogens by chemotaxis, opsonization, and lysis (58). Though, recent studies, including our investigations, revealed that complement has been largely underestimated in the past. It can modulate the adaptive immune response as T-cell activation can be directly induced by complement components or indirectly *via* complement-activated antigen-presenting cells such as macrophages (56, 59–61). Therefore, it might be possible that increased T-cell proliferation after treatment with PEGylated anti-TNFα agents is a result of complement activation. Noteworthy, immune cells themselves can function as local source of complement proteins (62). Macrophages and T-cells, which we used in our *in vitro* assay, are thus practically able to initiate full signaling through complement pathways with one exception: the formation of the membrane-attack complex involving C6–C9.

In summary, we demonstrate significant differences between the treatment with Cimzia^®^ and other anti-TNFα agents. We show that PEGylation of Remicade^®^ promotes immunostimulation and parasite control, an effect that we prove to be even more pronounced for Cimzia^®^. Our data indicate PEG-mediated complement activation to maintain T-cell activation, effector function, and parasite killing in hMDMs in the absence of sTNFα. Further examinations need to follow this study to determine detailed molecular mechanism of complement activation by PEG and its supportive role for *Lm* infection control. Considering that reactivation of a latent infection or a higher susceptibility for a new infection with *Leishmania* is a severe adverse effect of immunosuppressive anti-TNFα treatment, our findings contribute to a better understanding of the effectiveness of different TNFα blockers and will be helpful for the assessment of immunosuppressive anti-TNFα agents. Based on our results, we propose that anti-TNFα therapy using Cimzia^®^ may be advantageous for patients living in high-incidence areas of leishmaniasis.

## Author Contributions

KA and CF contributed equally to this work. KA, PC, ZW, GR, and GZ contributed conception and design of the study. KA, CF, HK, and AG performed and analyzed experiments. KA, KB, and GZ wrote the manuscript. All authors reviewed the data and edited and approved the final version of the manuscript.

## Conflict of Interest Statement

The authors declare that the research was conducted in the absence of any commercial or financial relationships that could be construed as a potential conflict of interest.
