# Supplementary material for: Anti-Tumor Necrosis Factor α Therapeutics Differentially Affect Leishmania Infection of Human Macrophages
Source: Front Immunol. 2018 Jul 31;9:1772. doi: 10.3389/fimmu.2018.01772 (PMC6079256; doi:10.3389/fimmu.2018.01772)
Supplement: Supplementary file 1 [file Data_Sheet_1.PDF]

Supplemental Data

Table S1: Fluorescent antibodies used for flow cytometry

Antibody	Clone	Company
Goat F(ab')2 anti-human IgG Fc RPE	polyclonal	Dianova
Mouse anti-human CD3 PB	UCHT1	BD Pharmingen
Mouse anti-human CD4 APC-Cy7	OKT4	BioLegend
Mouse anti-human CD8 APC-Cy7	SK1	BioLegend
Mouse anti-human CD16 APC	3G8	BioLegend
Mouse anti-human CD32 FITC	3D3	BD Pharmingen
Mouse anti-human CD45RO PE	UCHL1	BD Pharmingen
Mouse anti-human CD64 APC	10.1	BioLegend
Mouse anti-human granulysin A647	DH2	BioLegend
Mouse anti-human granzyme A A647	CB9	BioLegend
Mouse anti-human granzyme B A647	GB11	BioLegend
Mouse anti-human mTNFα PE	Mab11	BD Pharmingen
Mouse anti-human mTNFR1 APC	16803	R&D Systems
Mouse anti-human mTNFR2 APC	22235	R&D Systems
Mouse anti-human perforin APC	dG9	BioLegend

Figure S1

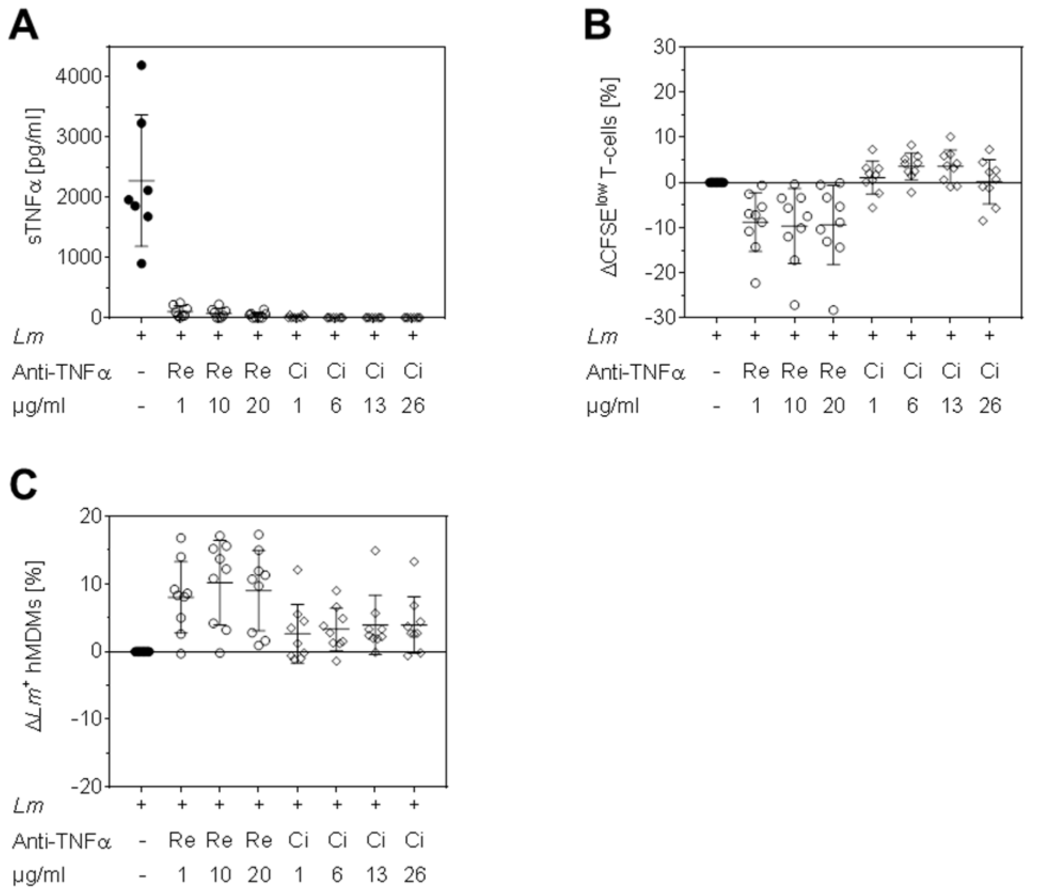

Figure S1: **Titration of Cimzia® or Remicade®.** Different concentrations of Cimzia® (Ci ◊) or Remicade® (Re ○) were added 24 hours after *Lm* infection and hMDMs were incubated in the presence of CFSE-labeled autologous PBLs. (A) The concentration of sTNFα was measured by ELISA after 7 days. T-cell proliferation (B) and the number of *Lm*-infected hMDMs (C) were analyzed by flow cytometry. Data are shown as differences by subtracting values of the respective controls for each donor and condition. 3 separate experiments were performed of which results are presented as mean ± SD ($n \geq 7$).

Figure S2

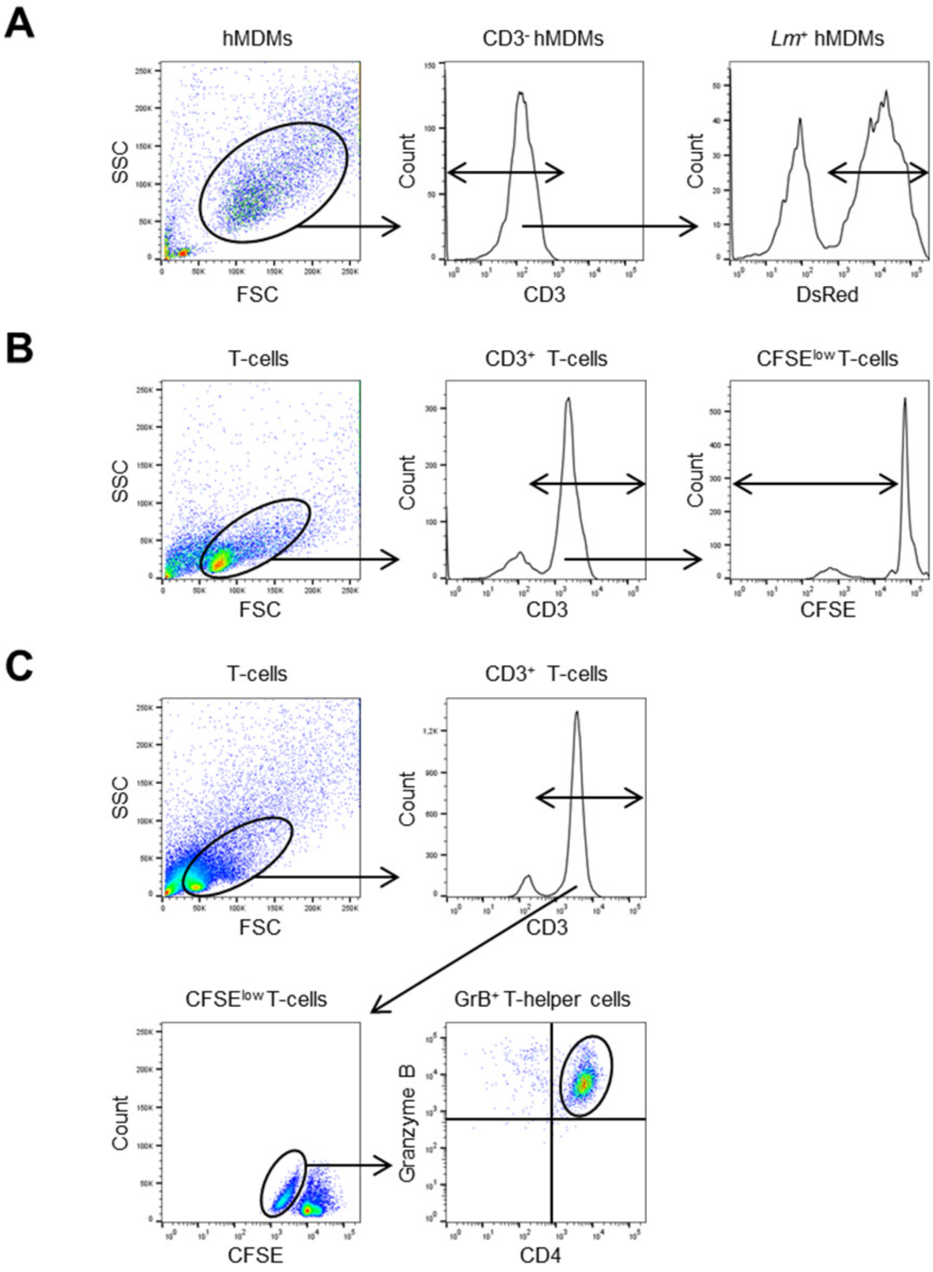

Figure S2: Gating strategies. (A) *Lm* infection rate: Macrophages were gated according to their SSC/FSC properties and $CD3^+$ T-cells were excluded using anti-CD3 Ab co-staining. The MFI was determined, where required, for surface expression of specific markers. The percentage of Lm^+ hMDMs was assessed using *Lm* that expressed EGFP or DsRed. (B) T-cell proliferation: $CD3^+$ T-cells were gated according to their SSC/FSC properties and anti-CD3 Ab co-staining. If necessary, surface

marker expression was assessed using the MFI. Proliferating T-cells were determined by the reduction of CFSE (CFSE^{low}). (C) Cytolytic protein expression in T-cells exemplarily shown for granzyme B (GrB): Fixed T-cells were defined by their size (SSC/FSC) and anti-CD3 Ab staining. Amongst proliferating T-cells (CFSE^{low}) the percentage of GrB and CD4 double-positive T-helper cells was assessed.

Figure S3

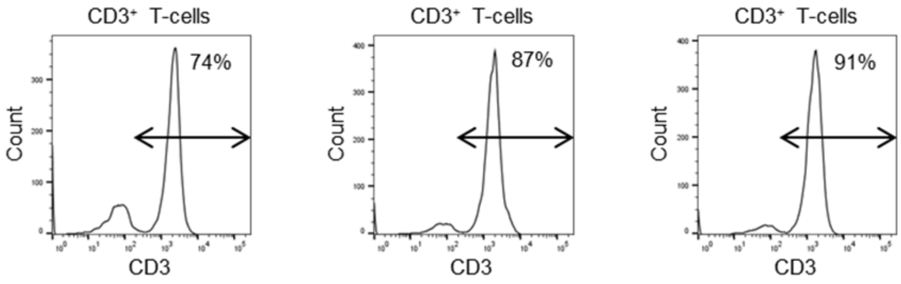

Figure S3: PBLs comprise approximately 70-90% T-cells. CD3^{+} T-cells were gated as described in **Figure S2**.

Figure S4

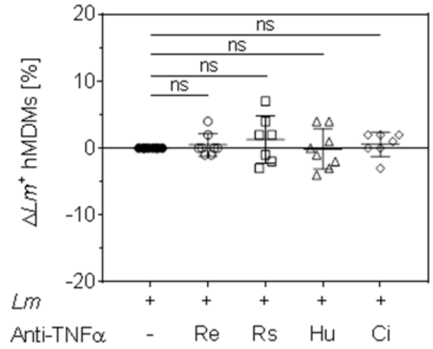

Figure S4: *Lm* infection rates in hMDMs after sTNFα blockade in the absence of PBLs. Macrophages were infected with *Lm*. 24 hours post-infection, the TNFα inhibitors Remicade® (Re ○), Remsima® (Rs □), Humira® (Hu Δ) and Cimzia® (Ci ◊) were added and cells were incubated in the absence of PBLs. After 7 days, the number of infected hMDMs was determined by flow cytometry. Values of the respective controls were subtracted for each donor and condition. Data are presented as mean ± SD ($n \geq 7$) and were obtained in 4 independent experiments. Statistical analysis was carried out using the Wilcoxon signed-rank test. ns $P > .05$.

Figure S5

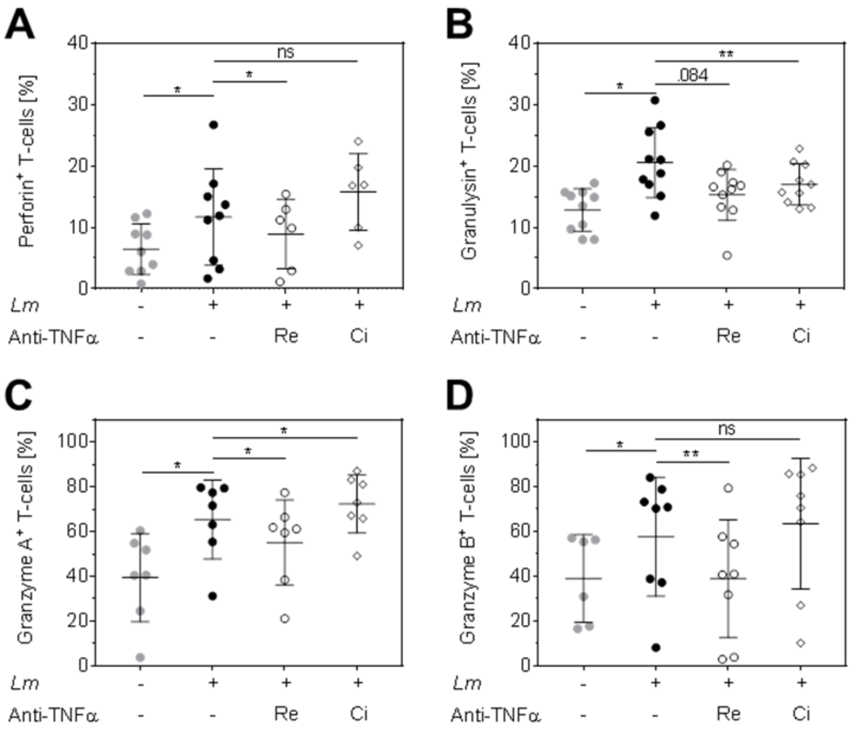

Figure S5: Intracellular expression of cytolytic proteins in proliferating CD4+ T-cells differs after sTNFα neutralization by Remicade® or Cimzia®. Non-infected (●) or *Lm*-infected (●) hMDMs were co-incubated with CFSE-labeled autologous PBLs and sTNFα was neutralized by Remicade® (Re ○) or Cimzia® (Ci ◊). Proliferating CD4+ T-cells expressing perforin (A), granulysin (B), granzyme A (C) or granzyme B (D) were detected using anti-CD3 and anti-CD4 Ab co-staining in intracellular flow cytometry 7 days after infection. Data are presented as mean ± SD ($n \geq 6$) and were obtained in at least 3 independent experiments. To analyze statistical significance, the Wilcoxon signed-rank test was performed.*$P < .05$; **$P < .01$.

Figure S6

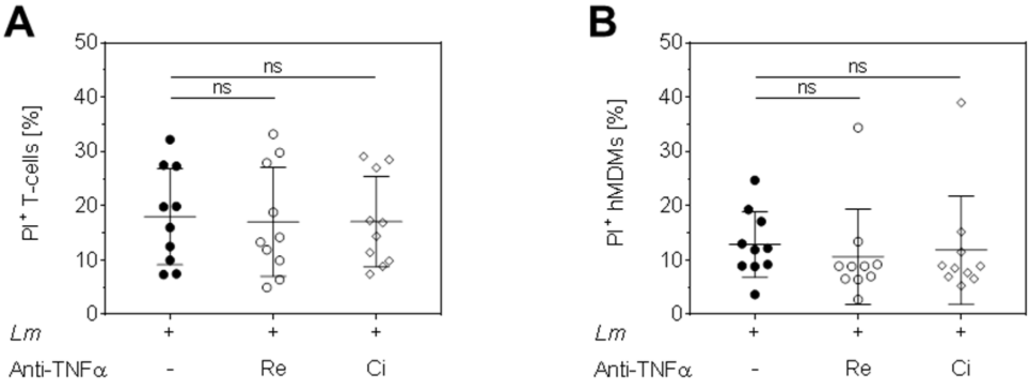

Figure S6: Treatment with Remicade® or Cimzia® has no impact on cell viability of T-cells or hMDMs. Macrophages were infected with *Lm* (●). 24 hours post-infection Remicade® (Re ○) and Cimzia® (Ci ◊) were added and cells were co-incubated with CFSE-labeled autologous PBLs. 7 days after infection, the percentage of PI^+ T-cells (A) and PI^+ hMDMs (B) was determined by anti-CD3 Ab co-staining in flow cytometry. Data are presented as mean ± SD (*n*=10) and were obtained in 4 independent experiments. The Wilcoxon signed-rank test was performed to evaluate statistical significance. ns $P > .05$.
